# Supplementary material for: Isolation and Potential Biological Applications of Haloaryl Secondary Metabolites from Macroalgae
Source: Mar Drugs. 2019 Jan 22;17(2):73. doi: 10.3390/md17020073 (PMC6409842; doi:10.3390/md17020073)
Supplement: Supplementary file 1 [file marinedrugs-17-00073-s001.pdf]

Review

Isolation and Potential Biological Applications of Haloaryl Secondary Metabolites from Macroalgae

Ana Jesus [1], Marta Correia-da-Silva [1,2,*], Carlos Afonso [1,2], Madalena Pinto [1,2], and Honorina Cidade [1,2,*]

[1] Laboratório de Química Orgânica e Farmacêutica, Departamento de Ciências Químicas, Faculdade de Farmácia, Universidade do Porto, Rua de Jorge Viterbo Ferreira 228, 4050-313 Porto, Portugal; anaaimjesus@gmail.com (A.J.); cafonso@ff.up.pt (C.A.); madalena@ff.up.pt (M.P.)

[2] Centro Interdisciplinar de Investigação Marinha e Ambiental (CIIMAR), Universidade do Porto, Terminal de Cruzeiros do Porto de Leixões, Avenida General Norton de Matos s/n 4450-208 Matosinhos, Portugal

* Correspondence: m_correiadasilva@ff.up.pt (M.C.-d.-S.); hcidade@ff.up.pt (H.C.); Tel: +351220428688 (H.C.)

Table of Contents

Table S1. Haloaryl secondary metabolites isolated from macroalgae and biological activities.

Table S1. Haloaryl secondary metabolites isolated from macroalgae and biological activities.

Secondary Metabolite	Specie/Family	Biological Activity
**Red Alga (Phylo Rhodophyta)**		
**Family: Cystocloniaceae**		
**1–14**	*Rhodophyllis membranaceae* [5]	Cytotoxic and antifungal activity [5]<br>(**1**)<br>IC_{50} = 38 μM (HL-60)<br>IC_{50} = 69 μM (S. *cerevisiae)*<br>(**2**, **5**, **7–10**, **12–14**)<br>IC_{50} > 10 μM (similar for both activities tested)<br>(**3**)<br>IC_{50} = 78 μM (HL-60)<br>IC_{50} = >83 μM (S. *cerevisiae*)<br>(**4**)<br>IC_{50} = 61 μM (HL-60)<br>IC_{50} = 63 μM (S. *cerevisiae*)<br>(**6**)<br>IC_{50} = 28 μM (HL-60)<br>IC_{50} = 23 μM (S. *cerevisiae*)<br>(**11**)<br>IC_{50} = 49 μM (HL-60)<br>IC_{50} = 39 μM (S. *cerevisiae*)
**Family: Halymeniaceae**		
**15**	*Grateloupia elliptica* [6]	α-glucosidase inhibitory activity [6]<br>IC_{50} = 60.3 μM (*S. cerevisiae* α-glucosidase)<br>IC_{50} = 130.3 μM (*B. stearoyhermophilus* α-glucosidase)<br>IC_{50} = 4.2 mM and 5.0 mM (rat intestinal sucrase and maltase)
**16**	*Grateloupia elliptica* [6]	α-glucosidase inhibitory activity [6]<br>IC_{50} = 110.4 μM (*S. cerevisiae* α-glucosidase)<br>IC_{50} = 230.3 μM (*B. stearoyhermophilus* α-glucosidase)

		IC_{50} = 3.6 mM and 4.8 mM (rat intestinal sucrase and maltase)
**17. BDDE**	*Polyopes lancifolia* [7]	α-glucosidase inhibitory activity [7]<br>IC_{50} = 0.098 and 0.120 μM (*S. cerevisiae* and *B. stearothermophilus*)<br>IC_{50} = 1.00 and 1.20 mM (rat intestinal sucrase and maltase)<br><br>Antifungal activity [14]<br>% inhibition = 80.0 ± 7.2; 77.1 ± 5.3; 75.0 ± 8.5; 67.7 ± 5.9 (*V. mali, F. graminearum, C. diplodiella* and *C. gloeosporioides)*<br><br>Antitumor activity [16]<br>IC_{50} = 13.9 μg/mL (K562)<br><br>Anti-angiogenesis (HUVEC) [13]
**Family: Lithothamniaceae**		
**18. Lithothamnin A**	*Lithothamnion fragilissimum* [17]	Antitumor activity [17]<br>IC_{50} = 9.5 μM (LOX)<br>IC_{50} = 7.6 μM (SNB-19)<br>IC_{50} = 7.6 μM (OVCAR-3)<br>IC_{50} = 19.0 μM (COLO-205)<br>IC_{50} = 19.0 μM (MOLT-4)
**Family: *Rhodomelaceae***		
***Callophycus* genus**		
**19–22**	*Callophycus sp.* [3]	----
***Laurencia* genus**		
**23–26**	(**23–26**) *Laurencia brongniartii* [18]<br>(**24–26**) *Laurencia decumbens* [20]<br>(**24**, **25**) *Laurencia similis* [19]<br>(**24–26**) *Laurencia complanata* [21]<br>(**23**) *Laurencia sp.* [22]	Antitumor activity (L1210)[18]<br>ID_{50} of 3.6 μg/mL (**25**)<br><br>Antimicrobial activity for *B. subtillus* and *S. cerevisiae* at 100 μg [18] (Ø inhibition = 16mm and 14mm respectively) (**25**)<br><br>Antibacterial activity [22]<br>MIC = 300 μg/mL (*Staphylococcus sp.*) (**24**)<br><br>Antimicrobial activity [21]<br>Ø inhibition = 11mm; 12mm; 12mm and 8.5 mm (*B. cereus*, *S. aureus*, *S. pneumoniae* and *C. albicans*) (**25**)
**27–30**	(**27–29**) *Laurencia similis* [19]	----

	(**30**) *Laurencia decumbens* [20]	
**31–33**	*Laurencia brongniartii* [23,24]	----
**34–39**	*Laurencia brongniartii* [23]	----
**40**	*Laurencia brongniartii* [23]	----
**41–43**	*Laurencia brongniartii* [23]	
**44–49**	*Laurencia brongniartii* [23]	----
**50**	*Laurencia brongniartii* [24]	----
**51**, **52**	*Laurencia brongniartii* [24]	Cytotoxic against [24]<br>(**51**) HT-29 and P-388 cell lines<br>(**52**) P-388 cell line
**53**	*Laurencia similis* [25]	----
**54. Cupalaurenol**	*Laurencia sp.* [22]	Antibacterial activity [22]<br>MIC = 125, 125, 125 and 200 μg/mL (*S. aureus*, *Staphylococcus sp.*, *Salmonella sp.* and *V. cholerae*)
**55. Laurinterol**	*Laurencia okamurai* [26]<br>*Laurencia microcladia* [27]<br>*Laurencia tristicha* [28]	Antitumor activity [27]<br>IC_{50} = 128.3 μM (K562)<br>IC_{50} = 67.2 μM (MC7)<br>IC_{50} =76.6 μM (PC3)<br>IC_{50} = 83.9 μM (HeLa)<br>IC_{50} = 74.6 μM (A431)<br>IC_{50} = 165.8 μM (CHO)
**56. Isolaurinterol**	*Laurencia okamurai* [26]<br>*Laurencia tristicha* [28]	----
**57. Dibromophenol**<br>**58. Neolaurinterol**	(**57, 58**) *Laurencia okamurai* [26]<br>(**57**) *Laurencia tristicha* [28]	----
**59. Aplysin**<br>**60. Aplysinol**<br>**61**	(**59–61**) *Laurencia okamurai* [26]<br>(**60**) *Laurencia tristicha* [28]	----
**62**	*Laurencia okamurai* [26]	----
**63. Allolaurinterol**	*Laurencia okamurai* [26]	----
**64–66**	*Laurencia okamurai* [26]	----
**67. Bromolaurenisol**	*Laurencia microcladia* [27]	Antitumor activity [27]<br>IC_{50} = 112.7 μM (K562)<br>IC_{50} = 78.3 μM (MC7)

		IC_{50}=92.4 µM (PC3)<br>IC_{50}= 105.8 µM (HeLa)<br>IC_{50}= 81.6 µM (A431)<br>IC_{50}= > 200 µM (CHO)
**68**	*Laurencia tristicha* [28]	-----
**69**	*Laurencia tristicha* [28]	-----
**70**	*Laurencia microcladia* [27]<br>*Laurencia tristicha* [28]	Antitumor activity [27]<br>IC_{50}= 153.5 µM (A549)
**71**	*Laurencia sp.* [29]	----
**72–74**	*Laurencia similis* [30]	PTP1B inhibitory activity [30]<br>IC_{50}= 102 µg/mL (**72**)<br>IC_{50}= 65.3 µg/mL (**73**)<br>IC_{50}= 69.8 µg/mL (**74**)
**5**	*Laurencia similis* [30]	PTP1B inhibitory activity [30]<br>IC_{50}= 2.66 µg/mL
**76**	*Laurencia similis* [30]	PTP1B inhibitory activity [30]<br>IC_{50}= 2.97 µg/mL
	***Odonthalia* genus**	
**17. BDDE**	*Odonthalia corymbifera* [8]	Inhibition of yeast α-glucosidase reaction [8]<br>IC_{50}= 0.098 µM
**77. Lanosol**	*Odonthalia corymbifera* [8]	α-glucosidase inhibitory activity [31]<br>IC_{50}= 89 µM (yeast α-glucosidase)<br>IC_{50}= 2.4 and 2.5 mM (rat- intestinal sucrase and maltase)<br>Antimicrobial activity (ICL inhibitors) [32]<br>IC_{50}= 92.6 ± 5.8 µM
**78-80**	*Odonthalia corymbifera* [31]	α-glucosidase inhibitory activity [31]<br>IC_{50}= 25 µM (yeast α-glucosidase) (**78**)<br>IC_{50}= 3.5 and 3.1 mM (rat intestinal sucrase and maltase α-glucosidase) (**78**)<br>IC_{50}= 53 µM (yeast α-glucosidase) (**79**)
**81**	*Odonthalia corymbifera* [31]	α-glucosidase inhibitory activity [31]<br>IC_{50}= 170 µM (yeast α-glucosidase)

		Antimicrobial activity (ICL inhibitors) [32]<br>IC_{50} = 125.6 ± 8.6 μM
**82**	*Odonthalia corymbifera* [32]	Antimicrobial activity (ICL inhibitors) [32]<br>IC_{50} = 116.1 ± 7.3 μM
**83, 84**	*Odonthalia corymbifera* [32]	Antimicrobial activity (ICL inhibitors) [32]<br>(**83**) IC_{50} = 2.1 ± 0.1 μM<br>(**84**) IC_{50} = 2.8 ± 0.2 μM
**85. BDDPM**	*Odonthalia corymbifera* [32]	Antimicrobial activity (ICL inhibitor) [32]<br>IC_{50} = 2.0±0.1 μM
**86. Odonthadione**	*Odonthalia corymbifera* [33]	DPPH and ABTS radical scavenging activity [33]<br>EC_{50} = 24.7 μM (DPPH) EC_{50} = 17.3 μM (ABTS)<br><br>Tyrosinase inhibition [33]<br>IC_{50} = 17.3 μM
**87. Odonthalol**	*Odonthalia corymbifera* [33]	DPPH and ABTS radical scavenging activity [33]<br>EC_{50} = 13.5 μM (DPPH)<br>EC_{50} =6.7 μM (ABTS)<br><br>Tyrosinase inhibition [33]<br>IC_{50} = 31.0 μM
	***Osmundaria* genus**	
**77. Lanosol**	*Osmundaria colensoi* [34]	---
**81**	*Osmundaria colensoi* [34]	Antibacterial activity (*Mycobacterium smegmatis*) [34]<br>IC_{50} = 7.8 μM
**88. Colensolide A**	*Osmundaria colensoi* [34]	----
**89**	*Osmundaria colensoi* [34]	----
**90**	*Osmundaria colensoi* [34]	Antibacterial activity (*Mycobacterium smegmatis*) [34]<br>IC_{50} = 26.2 μM<br><br>Antitumor activity [34]<br>IC_{50} = 8.0 μM (HL-60)
**91. Rhodomelol**	*Osmundaria colensoi* [34]	Antibacterial activity (*Mycobacterium smegmatis*) [34]<br>IC_{50} = 28.1 μM
	***Polysiphonia* genus**	
**77. Lanosol**	*Polysiphonia lanosa* [35]	Antitumor activity [35]

		IC_{50} = 18.3 ± 0.94 μM (DLD-1) and 20.4 ± 2.9 μM (HCT-116)
**81**	*Polysiphonia lanosa* [35]	Antitumor activity [35]<br>IC_{50} = 14.6 ± 3.1 μM (DLD-1) and 14.1 ± 2.5 μM (HCT-116)
**89**	*Polysiphonia lanosa* [35]	Antitumor activity [35]<br>IC_{50} = 30.9 ± 2.7 μM (DLD-1)
**92**, **93**	*Polysiphonia morrowii* [31]	α-glucosidase inhibitory activity [31]<br>IC_{50}= 100 μM (yeast α-glucosidase) (**92**)<br>IC_{50} = 3.6 and 4.8 mM (rat intestinal sucrase and maltase α-glucosidase) (**92**)<br>IC_{50} = > 1000 μM (yeast α-glucosidase) (**93**)
**94**, **95**	*Polysiphonia lanosa* [35]	Antitumor activity [35]<br>(**94**) IC_{50} = 13.5 ± 2.3 μM (DLD-1) and 2.51 ± 0.95 μM (HCT-116)<br>(**95**) IC_{50} = 12.4 ± 1.1 μM (DLD-1) and 1.32 ± 0.3 μM (HCT-116)
**96**	*Polysiphonia urceolata* [36]	DPPH radical scavenging activity [36]<br>IC_{50} = 16.11 ± 0.06 μM
**97**	*Polysiphonia urceolata* [36]	DPPH radical scavenging activity [36]<br>IC_{50} = 21.90 ± 0.1 μM
**98**	*Polysiphonia urceolata* [36]	DPPH radical scavenging activity [36]<br>IC_{50} = 9.67 ± 0.04 μM
**99**, **100**	*Polysiphonia urceolata* [37]	DPPH radical scavenging activity [37]<br>IC_{50} = 6.8 μM (**99**)<br>IC_{50} = 6.1 μM (**100**)
**101**	*Polysiphonia urceolata* [37]	DPPH radical scavenging activity [37]<br>IC_{50} = 8.1 μM
**102. Urceolatin**	*Polysiphonia urceolata* [38]	DPPH radical scavenging activity [38]<br>IC_{50} =7.9 μM
**103**	*Polysiphonia urceolata* [36]	DPPH radical scavenging activity [36]<br>IC_{50} = 19.64 ± 0.09 μM
**104. Urceolatol**	*Polysiphonia urceolata* [37]	DPPH radical scavenging activity [37]<br>IC_{50} = 15.1 μM
**105**, **106**	(**105**, **106**) *Polysiphonia urceolata* [37]<br>(**106**) *Polysiphonia morrowii* [41]	DPPH radical scavenging activity [37]<br>IC_{50} = 20.3 μM (**105**)<br>IC_{50} = 35.8 μM (**106**)<br>Antiviral activity [41]

		(**106**)<br>EC_{50} = 45.0 ± 9.1 μM (IHNV) and 57.0 ± 10.6 μM (IPNV)
**107. (±) Polysiphenol**	*Polysiphonia ferulacea* [39]	------
**108**	*Polysiphonia morrowii* [41]	Antiviral activity [41]<br>EC_{50} = 27.0 ± 6.3 μM (IHNV) and 22.0 ± 0.6 μM (IPNV)
	***Rhodomela* genus**	
**17. BDDE**	*Rhodomela confervoides* [9]	DPPH and ABTS radicals scavenging activity [11]<br>IC_{50} = 17.61 ± 0.08 μM (DPPH)<br>TEAC = 3.05 ± 0.13 nM (ABTS)<br><br>Antibacterial activity [15]<br>MIC = 35 μg/mL (*Staphylcoccus epidermidis* ATCC12228)<br>MIC = 70 μg/mL (*Staphylcoccus aureus* ATCC29213, *Staphylcoccus aureus* 02-60, *Pseudomonas aeruginosa* ATCC27853 and *Pseudomonas aeruginosa* 02-29)<br>MIC = 140 μg/mL (*Staphylcoccus epidermidis* 02-4 and *Escherichia coli* ATCC25922)<br>MIC < 140 μg/mL (*Escherichia coli* 02-26)<br><br>PTP1B inhibitory activity [12]<br>IC_{50} = 1.5 μmol/mL<br><br>Antitumor activity [42]<br>IC_{50} = 4.19 μg/mL (KB)<br>IC_{50} = 7.94 μg/mL (Bel 7402)<br>IC_{50} = > 10 μg/mL (A549)<br>IC_{50} = 7.41 μg/mL (HELF)
**77. Lanosol**	*Rhodomela confervoides* [9,45]	DPPH and ABTS radical scavenging activity [45]<br>IC_{50} = 42.33 ± 0.25 μM (DPPH)<br>TEAC = 1.56 ± 0.02 mM (ABTS)
**81**	*Rhodomela confervoides* [9,45]	DPPH and ABTS radical scavenging activity [45]<br>IC_{50} = 40.50 ± 0.20 μM (DPPH)<br>TEAC = 1.62 ± 0.03 mM (ABTS)
**83**	*Rhodomela confervoides* [45]	DPPH and ABTS radical scavenging activity [45]<br>IC_{50} = 32.01 ± 0.12 μM<br>TEAC = 1.09 ± 0.01 mM

		DPPH and ABTS radical scavenging activity [11]<br>IC_{50} = 14.32 ± 0.12 μM (DPPH)<br>TEAC = 3.00 ± 0.13 mM (ABTS)<br><br>Antibacterial activity [15]<br>MIC = 70 μg/mL (*S. aureus* ATCC29213, Staphylcoccus aureus 02-60, Staphylcoccus epidermidis ATCC12228 and Staphylcoccus epidermidis 02-4)
**84**	*Rhodomela confervoides* [9]	DPPH and ABTS radical scavenging activity [11]<br>(**84**)<br>IC_{50} = 19.60 ± 0.11 μM (DPPH)<br>TEAC = 3.16 ± 0.14 mM (ABTS)
**85. BDDPM**	*Rhodomela confervoides* [9]	DPPH and ABTS radical scavenging activity [11]<br>IC_{50} = 16.91 ± 0.10 μM<br>TEAC = 3.18 ± 0.13 mM<br><br>PTP1B inhibitory activity [12]<br>IC_{50} = 2.4 μmol/L<br><br>Antibacterial activity [15]<br>MIC = 140 μg/mL (S. aureus ATCC29213, *S. aureus* 02-60, *S. epidermidis* ATCC12228, *S. epidermidis 02-4*, *Escherichia coli ATCC25922*, *P. aeruginosa* ATCC27853 and *Pseudomonas aeruginosa* 02-29*)*<br><br>Antitumor activity [44]<br>IC_{50} = 17.63 μg/mL (HeLa)<br>IC_{50} = 11.37 μg/mL (RKO)<br>IC_{50} = 10.58 μg/mL (HCT-116)<br>IC_{50} = 8.7 μg/mL (Bel 7402)<br>IC_{50} = 23.69 μg/mL (U87)<br>IC_{50} = 30.15 μg/mL (HUVEC)
**90**	*Rhodomela confervoides* [45]	DPPH and ABTS radical scavenging activity [45]<br>IC_{50} = 7.62 ± 0.01 μM (DPPH)<br>TEAC = 3.45 ± 0.12 mM (ABTS)
**92**	*Rhodomela confervoides* [45]	----
**94**	*Rhodomela confervoides* [45]	DPPH and ABTS radical scavenging activity [45]<br>IC_{50} = 38.42 ± 0.23 μM

		TEAC = 1.36 ± 0.01 mM
**109,110. BPN**	*Rhodomela confervoides* [9]	Antibacterial activity [15]<br>(**109**)<br>MIC = 140 μg/mL (*S. aureus* ATCC29213, *S. aureus* 02-60 and *S. epidermidis* ATCC12228<br>MIC < 140 μg/mL (*E. coli* ATCC25922 and *P. aeruginosa* ATCC27853<br><br>Antibacterial activity [15]<br>(**110**)<br>MIC = 70 μg/mL (*S. aureus* ATCC29213)<br>MIC = 140 μg/mL (*S. aureus* 02-60, *S. epidermidis* ATCC12228, *S. epidermidis 02-4 and Escherichia coli ATCC25922*)<br><br>DPPH and ABTS radical scavenging activity [11]<br>(**110**)<br>IC_{50} = 13.81 ± 0.08 μM (DPPH)<br>TEAC = 2.78 ± 0.12 mM (ABTS)<br><br>PTP1B inhibitory activity [12]<br>(**110**)<br>IC_{50} = 0.84 μM
**111**	*Rhodomela confervoides* [9]	----
**112–114**	(**112**) *Rhodomela confervoides* [9]<br>(**112–114**) *Rhodomela confervoides* [42]	Antitumor activity [42]<br>(**112**)<br>IC_{50} = 3.09 μg/mL (KB)<br>IC_{50} = 3.18 μg/mL (Bel 7402)<br>IC_{50} = 3.54 μg/mL (A549)<br>IC_{50} = 6.39 μg/mL (HELF)<br>(**113**)<br>IC_{50} = 6.26 μg/mL (KB)<br>IC_{50} = 3.33 μg/mL (Bel 7402)<br>IC_{50} = 7.08 μg/mL (A549)<br>IC_{50} = 2.65 μg/mL (HELF)<br>(**114**)<br>IC_{50} = 8.71 μg/mL (KB)<br>IC_{50} = 5.36 μg/mL (Bel 7402)

		IC50 = 7.56 μg/mL (A549)<br>IC50 = > 10 μg/mL (HELF)
**115**	*Rhodomela confervoides* [43]	----
**116**	*Rhodomela confervoides* [43]	----
**117**	*Rhodomela confervoides* [43]	----
**118**	*Rhodomela confervoides* [43]	----
**119–121**	*Rhodomela confervoides* [43,45]	Antitumor activity [43]<br>(**119**)<br>IC50 = 19.7 μM (A549)<br>IC50 = 19.9 μM (A2780)<br>IC50 = 19.4 μM (Bel7402)<br>IC50 = 20.2 μM (BGC-823)<br>IC50 = 15.4 μM (HCT-8)<br>(**120**)<br>IC50 = 14.7 μM (A549)<br>IC50 = 9.4 μM (A2780)<br>IC50 = 14.8μM (Bel7402)<br>IC50 = 24.0 μM (BGC-823)<br>IC50 = 14.6 μM (HCT-8)<br>(**121**)<br>IC50 = 18.5 μM (A549)<br>IC50 = 20.8 μM (A2780)<br>IC50 = 20.4 μM (Bel7402)<br>IC50 = 19.1μM (BGC-823)<br>IC50 = 18.8 μM (HCT-8)<br><br>DPPH and ABTS radical scavenging activity [45]<br>(**119**)<br>IC50 = 30.91 ± 0.12 μM (DPPH)<br>TEAC = 1.98 ± 0.01 mM (ABTS)
**122**	*Rhodomela confervoides* [43]	Antitumor activity [43]<br>IC50 = 14.5 μM (A549)<br>IC50 = > 16.9 μM (A2780)<br>IC50 = 13.5 μM (Bel7402)<br>IC50 = 15.1 μM (BGC-823)

		IC_{50} = 12.1 μM (HCT-8)
**123. BDB**	*Rhodomela confervoides* [11,12]	DPPH and ABTS radical scavenging activity [11]<br>IC_{50} = 8.90 ± 0.04 μM (DPPH)<br>TEAC = 3.58 ± 0.13 mM (ABTS)<br>PTP1B inhibitory activity[12,61]<br>IC_{50} = 1.7 μmol/L
**124**	*Rhodomela confervoides* [45]	DPPH and ABTS radical scavenging activity [45]<br>IC_{50} = 7.43 ± 0.10 μM (DPPH)<br>TEAC = 2.11 ± 0.04 mM (ABTS)
**125–132**	*Rhodomela confervoides* [45]	DPPH and ABTS radical scavenging activity [45]<br>(**125**)<br>IC_{50} = 20.47 ± 0.07 μM (DPPH)<br>TEAC = 1.87 ± 0.02 mM (ABTS)<br>(**126**)<br>IC_{50} = 19.84 ± 0.06 μM (DPPH)<br>TEAC = 2.87 ± 0.11 mM (ABTS)<br>(**127**)<br>IC_{50} = 50.58 ± 0.23 μM (DPPH)<br>TEAC = 1.60 ± 0.04 mM (ABTS)<br>(**128**)<br>IC_{50} = 26.28 ± 0.21 μM (DPPH)<br>TEAC = 2.35 ± 0.02 mM (ABTS)<br>(**129**)<br>IC_{50} = 30.24 ± 0.20 μM (DPPH)<br>TEAC = 2.07 ± 0.12 mM (ABTS)<br>(**130**)<br>IC_{50} = 58.15 ± 0.39 μM (DPPH)<br>TEAC = 1.32 ± 0.02 mM (ABTS)<br>(**131**)<br>IC_{50} = 9.52 ± 0.04 μM (DPPH)<br>TEAC = 2.06 ± 0.08 mM (ABTS)<br>(**132**)<br>IC_{50} = 50.31 ± 0.34 μM (DPPH)

		TEAC = 1.86 ± 0.02 mM (ABTS)
**133**	*Rhodomela confervoides* [45]	DPPH and ABTS radical scavenging activity [45]<br>IC_{50} = 8.72 ± 0.05 μM (DPPH)<br>TEAC = 3.68 ± 0.12 mM (ABTS)
**134**, **135**	*Rhodomela confervoides* [45]	DPPH and ABTS radical scavenging activity [45]<br>(**134**)<br>IC_{50} = 18.62 ± 0.08 μM (DPPH)<br>TEAC = 2.11 ± 0.11 mM (ABTS)<br>(**135**)<br>IC_{50} = 50.87 ± 0.32 μM (DPPH)<br>TEAC = 1.63 ± 0.01 mM (ABTS)
**136**	*Rhodomela confervoides* [11]	DPPH and ABTS radicals scavenging activity [11]<br>IC_{50} = 13.60 ± 0.03 μM (DPPH)<br>TEAC = 3.21 ± 0.13 mM (ABTS)
**137–139**	*Rhodomela confervoides* [11]	DPPH and ABTS radicals scavenging activity [11]<br>(**137**)<br>IC_{50} = 15.90 ± 0.09 μM (DPPH)<br>TEAC = 2.68 ± 0.11 mM (ABTS)<br>(**138**)<br>IC_{50} = 18.50 ± 0.18 μM (DPPH)<br>TEAC = 2.21 ± 0.12 mM (ABTS)<br>(**139**)<br>IC_{50} = 5.22 ± 0.04 μM (DPPH)<br>TEAC = 2.87 ± 0.10 mM (ABTS)
**140**, **141**	*Rhodomela confervoides* [11]	DPPH and ABTS radicals scavenging activity [11]<br>(**140**)<br>IC_{50} = 5.43 ± 0.02 μM (DPPH)<br>TEAC = 2.31 ± 0.11 mM (ABTS)<br>(**141**)<br>IC_{50} = 5.70 ± 0.03 μM (DPPH)<br>TEAC = 2.14 ± 0.08 mM (ABTS)
**142**, **143**	*Rhodomela confervoides* [11]	DPPH and ABTS radicals scavenging activity [11]<br>(**142**)<br>IC_{50} = 23.60 ± 0.10 μM (DPPH)

		TEAC = 2.11 ± 0.04 mM (ABTS)<br>(**143**)<br>IC_{50} = 20.81 ± 0.08 μM (DPPH)<br>TEAC = 2.36 ± 0.08 mM (ABTS)
	***Symphyocladia* genus**	
**81**	*Symphyocladia latiuscula* [48]	PTP1B inhibitory activity [48]<br>IC_{50} = 39.0 ± 4.0 μmol/l
**144**	*Symphyocladia latiuscula* [31,49]	α-glucosidase inhibitory activity [31]<br>IC_{50} = 11 μM (yeast α-glucosidase)<br>IC_{50} = 4.2 mM and > 5.0 mM (rat intestinal sucrase and maltase)<br><br>DPPH radical scavenging activity [49]<br>IC_{50} = 7.5 μM
**145**, **146**	*Symphyocladia latiuscula* [31,47,50]	α-glucosidase inhibitory activity [31]<br>IC_{50} = 0.030 μM (yeast α-glucosidase) (**145**)<br>IC_{50} = 2.4 mM and 3.2 mM (rat intestinal sucrase and maltase) (**145**)<br><br>Aldose inhibitory activity [47]<br>IC_{50} = 0.40 μg/mL (**145**)<br>IC_{50} = 0.40 μg/mL (**146**)<br><br>DPPH radical scavenging activity [50]<br>(**145**)<br>IC_{50} = 8.1 μM<br><br>PTP1B inhibitory activity [48]<br>(**145**)<br>IC_{50} = 4.3 ± 0.1 μmol/L
**147**	*Symphyocladia latiuscula* [47]	Aldose inhibitory activity [47]<br>IC_{50} = 0.11 μmol/mL
**148**	*Symphyocladia latiuscula* [48]	DPPH radical scavenging activity [50]<br>IC_{50} = 10.2 μM<br><br>PTP1B inhibitory activity [48]<br>IC_{50} = 3.5 ± 0.2 μmol/L
**149–151**	*Symphyocladia latiuscula* [47,48]	Aldose inhibitory activity [47]<br>(**150**) IC_{50} = 1.15 μg/mL

		(**151**) IC_{50} = 0.25 µg/mL<br><br>DPPH radical scavenging activity [50]<br>(**149**)<br>IC_{50} = 8.5 µM<br>(**150**)<br>IC_{50} = 14.0 µM<br>(**151**)<br>IC_{50} = 24.7 µM<br><br>PTP1B inhibitory activity [48]<br>(**149**)<br>IC_{50} = 3.9 ± 0.2 µmol/L
**152**	*Symphocladia latiuscula* [48]	PTP1B inhibitory activity [48]<br>IC_{50} = > 50 µmol/L
**153**	*Symphocladia latiuscula* [48,50]	DPPH radical scavenging activity [50]<br>IC_{50} = 18.5 µM<br><br>PTP1B inhibitory activity [48]<br>IC_{50} = 25.6 ± 4.8 µmol/L
**154**	*Symphyocladia latiuscula* [50]	DPPH radical scavenging activity [50]<br>IC_{50} = 24.0 µM
**155**	*Symphyocladia latiuscula* [49]	DPPH radical scavenging activity [49]<br>IC_{50} = 8.5 µM
**156**	*Symphyocladia latiuscula* [31,50]	DPPH radical scavenging activity [50]<br>IC_{50} = 10.5 µM<br><br>Antifungal Activity [51]<br>MIC = 12.5 µg/mL (*C. albicans*)
**157**	*Symphyocladia latiuscula* [47]	DPPH radical scavenging activity [50]<br>IC_{50} = 24.0 µM
**158**	*Symphyocladia latiuscula* [46]	DPPH radical scavenging activity [46]<br>IC_{50} = 14.5 µg/mL
**159**	*Symphyocladia latiuscula* [46]	DPPH radical scavenging activity [46]<br>IC_{50} = 20.5 µg/mL
**160**	*Symphyocladia latiuscula* [51]	Antifungal Activity [51]

		MIC = > 100 μg/mL (*C. albicans*)
**161, 162**	*Symphyocladia latiuscula* [51]	Antifungal activity [51]<br>(**161**)<br>MIC = > 100 μg/mL (*C. albicans*)<br>(**162**)<br>MIC = 25 μg/mL (*C. albicans*)
**163**	*Symphyocladia latiuscula* [52]	Antifungal activity [52]<br>MIC = 37.5 μg/mL (*C. albicans*)
**164. SL-1**<br>**165. SL-2**	*Symphyocladia latiuscula* [53]	(**164-165**) Inhibitor of Taq DNA polymerase activity [53]
	***Vidalia* genus**	
**166. Vidalol A**	*Vidalia obtusaloba* [54]	Anti-inflammatory activity [54]<br>(inhibition of phospholipase A_2)
**167. Vidalol B**	*Vidalia obtusaloba* [54]	Anti-inflammatory activity [54]<br>(inhibition of phospholipase A_2)
	**Brown Alga (Phylo Phaeophyta)**	
	**Family: Chordariaceae**	
**17. BDDE**	*Leathesia nana* [10]	Antitumor activity [10]<br>IC_{50} = 0.0054 μM/mL (A549)<br>IC_{50} = 0.0180 μM/mL (BGC-823)<br>IC_{50} = 0.0046 μM/mL (MCF-7)<br>IC_{50} = 0.0074 μM/mL (Bel7402)<br>IC_{50} = 0.0059 μM/mL (HCT-8)
**77. Lanosol**	*Leathesia nana* [10]	----
**81**	*Leathesia nana* [10]	----
**83**	*Leathesia nana* [10]	Antitumor activity [10]<br>IC_{50} = > 0.0195 μM/mL (A549)<br>IC_{50} = 0.0086 μM/mL (BGC-823)<br>IC_{50} = 0.00214 μM/mL (MCF-7)<br>IC_{50} = > 0.0019 μM/mL (Bel 7402)<br>IC_{50} = > 0.0207 μM/mL (HCT-8)<br>Antitumor activity [56]<br>IC_{50} = > 10 μg/mL (A549)

		IC_{50} = 4.42 μg/mL (BGC-823)<br>IC_{50} = 9.0 μg/mL (MCF-8)<br>IC_{50} = 7.88 μg/mL (B16-L16)<br>IC_{50} = 5.28 μg/mL (HT-1080)<br>IC_{50} = > 10 μg/mL (A2780)<br>IC_{50} = > 10 μg/mL (Bel7402)<br>IC_{50} = 9.64 μg/mL (HCT-8)
**85. BDDPM**	*Leathesia nana* [10]	Antitumor activity [10]<br>IC_{50} = 0.0018 μM/mL (A549)<br>IC_{50} = 0.0038 μM/mL (BGC-823)<br>IC_{50} = 0.0027 μM/mL (MCF-7)<br>IC_{50} = > 0.0182 μM/mL (Bel 7402)<br>IC_{50} = > 0.0022 μM/mL (HCT-8)
**89**	*Leathesia nana* [10]	----
**94**	*Leathesia nana* [10]	-----
**110. BPN**	*Leathesia nana* [10]	Antitumor activity [10]<br>IC_{50} = > 0.0190 μM/mL (A549)<br>IC_{50} = 0.0046 μM/mL (BGC-823)<br>IC_{50} = 0.0034 μM/mL (MCF-7)<br>IC_{50} = 0.0055 μM/mL (Bel 7402)<br>IC_{50} = 0.0028 μM/mL (HCT-8)<br><br>Antitumor activity [56]<br>IC_{50} = > 10 μg/mL (A549)<br>IC_{50} = 2.40 μg/mL (BGC-823)<br>IC_{50} = 1.81 μg/mL (MCF-8)<br>IC_{50} = 1.75 μg/mL (B16-L16)<br>IC_{50} = 3.81 μg/mL (HT-1080)<br>IC_{50} = 3.76 μg/mL (A2780)<br>IC_{50} = 2.97 μg/mL (Bel7402)<br>IC_{50} = 1.46 μg/mL (HCT-8)<br><br>Moderate inhibitory activity against protein tyrosine kinase (PTK) [56]
**168**	*Leathesia nana* [10]	----

**169**	*Leathesia nana* [10]	----
**170**	*Leathesia nana* [10]	Antitumor activity [10]<br>IC_{50} = 0.0025 μM/mL (A549)<br>IC_{50} = 0.0088 μM/mL (BGC-823)<br>IC_{50} = 0.0027 μM/mL (MCF-7)<br>IC_{50} = 0.0048 μM/mL (Bel 7402)<br>IC_{50} = > 0.0168 μM/mL (HCT-8)<br><br>Antitumor activity [56]<br>IC_{50} = 1.49 μg/mL (A549)<br>IC_{50} = 5.21 μg/mL (BGC-823)<br>IC_{50} = 3.46 μg/mL (MCF-8)<br>IC_{50} = 4.25 μg/mL (B16-L16)<br>IC_{50} = 3.84 μg/mL (HT-1080)<br>IC_{50} = 1.58 μg/mL (A2780)<br>IC_{50} = 2.83 μg/mL (Bel7402)<br>IC_{50} = > 10 μg/mL (HCT-8)<br><br>Moderate inhibitory activity against protein tyrosine kinase (PTK) [56]
**171**	*Leathesia nana* [10]	----
**172**	*Leathesia nana* [10]	Antitumor activity [56]<br>IC_{50} = > 10 μg/mL (A549, BGC-823, B16-L16, A2780, Bel7402 and HCT-8)<br>IC_{50} = 8.27 μg/mL (MCF-8)<br>IC_{50} = 6.36 μg/mL (HT-1080)<br><br>Moderate inhibitory activity against protein tyrosine kinase (PTK) [56]
**173**	*Leathesia nana* [10]	----
**174**	*Leathesia nana* [10]	----
**175**	*Leathesia nana* [10]	----
**176–178**	*Leathesia nana* [10]	----
	**Family: Dictyotaceae**	
**179**	*Stypopodium flabeliforme* [57]	----
	**Green Alga (Phylo *Clorophyta*)**	
	**Family: Cladophoraceae**	
**180**	*Chaetomorpha bariretorsa* [58]	-----

	Family: Dichotomosiphonaceae	
**181. Avrainvilleol**	*Avrainvillea nigricans* [60]	Antimicrobial activity [60]<br>Ø inhibition = 10 mm at 25 μg; 12 mm at 50 μg and 14 mm at 100 μg for *Bacillus subtillus*<br>Ø inhibition = 9 mm at 200 μg for *Serratia marcesens*<br>Ø inhibition = 10 mm at 25 μg; 12 mm = 50 μg; 16 mm = 100 μg for *Staphylococcus aureus*<br><br>Antitumor activity (KB) [60]<br>ID_{50} = 10-100 μg/mL
**182. 5'-Hydroxyisoavrainvilleol**	*Avrainvillea nigricans* [60]	Antimicrobial activity[60]<br>Ø inhibition = 9 mm at 25 μg; 10 mm at 50 μg and 11 mm at 100 μg for *Bacillus subtillus*<br>Ø inhibition = 10 mm at 100 μg for *Staphylococcus aureus*
**183**	*Avrainvillea nigricans* [60]	Antitumor activity (KB) [60]<br>ID_{50} = 8.9 μg/mL<br><br>Antimicrobial activity [60]<br>Ø inhibition = 8 mm at 25 μg; 10 mm at 50 μg and 12 mm at 100 μg for *Bacillus subtillus*<br>Ø inhibition = 8 mm at 25 μg; 11 mm at 50 μg and 13 mm at 100 μg for *Staphylococcus aureus*<br>Ø inhibition = 8 mm at 100 μg; 9 mm = 200 μg for *Pseudomonas aeruginosa*<br>Ø inhibition = 10 mm at 200 μg for *Escherichia coli*<br>Ø inhibition = 9 mm at 100 μg; 13 mm = 200 μg for *Serratia marcesens*<br>Ø inhibition = 8 mm at 200 μg for *Candida albicans*
**184. Rawsonol**	*Avrainvillea rawsoni* [59]	HMG-CoA reductase inhibitory activity [59]<br>IC_{50} = 5 μM
